# Decreased blood CD4+ T lymphocyte helps predict cognitive impairment in patients with amyotrophic lateral sclerosis

**DOI:** 10.1186/s12883-021-02185-w

**Published:** 2021-04-12

**Authors:** Yuan Yang, Dengji Pan, Zhenxiang Gong, Jiahui Tang, Zehui Li, Fengfei Ding, Mao Liu, Min Zhang

**Affiliations:** 1 Department of Neurology, Tongji Hospital, Tongji Medical College, Huazhong University of Science and Technology, Jie Fang Avenue 1095, 430030 Wuhan, Hubei PR China; 2 Department of Pharmacology, School of Basic Medical Sciences, Fudan University, Shanghai, China

**Keywords:** Amyotrophic lateral sclerosis, Cognitive impairment, ECAS, Peripheral blood lymphocyte subsets

## Abstract

**Background:**

ALS patients have changed peripheral immunity. It is unknown whether peripheral immunity is related to cognitive dysfunction in ALS patients.

**Objective:**

To explore the relationship between the peripheral blood lymphocyte subsets and the cognitive status in ALS patients.

**Methods:**

Among 81 ALS patients, we compared the demographic, clinical, and peripheral levels of total T lymphocyte, CD4+ T lymphocyte, CD8+ T lymphocyte, B lymphocyte, and NK cell between those with cognitive impairment (ALS-ci) and those without (ALS-nci). The cognitive status was evaluated via the Chinese version of the Edinburgh cognitive and behavioral screen (ECAS). Significant predictors of cognitive impairment in univariate logistic regression analysis were further examined using multivariate logistic regression analysis.

**Results:**

39.5% of all ALS patients had cognitive impairment. The ALS-ci group had shorter education time, older age at both symptom onset and testing, longer disease duration, and lower levels of peripheral total, CD4+, and CD8+ T lymphocyte and B lymphocyte than the ALS-nci group. Frequency of behavioral impairment did not differ between the two groups. While parameters with significant differences identified by group comparison were also significant predictors of cognitive impairment in univariate logistic regression analysis except the level of B lymphocyte, only older age at testing, education time less than 9 years, and lower level of CD4+ T lymphocyte remained significant in multivariate logistic regression analysis. The predictive model combining these three parameters had an area under the receiver operating characteristic curve value of 0.842 with a sensitivity of 90.6% and a specificity of 67.3%.

**Conclusion:**

In Chinese ALS patients, blood CD4+ T lymphocyte might help evaluate cognitive impairment along with age and education level.

**Supplementary Information:**

The online version contains supplementary material available at 10.1186/s12883-021-02185-w.

## Introduction

Amyotrophic lateral sclerosis (ALS) is a fatal and progressive neurodegenerative disease characterized by loss of upper and lower motor neurons [1]. While ALS was initially considered to selectively involve the motor system, evidence suggests that ALS could involve multiple systems including the cognitive function [2]. Nearly half of ALS patients have mild cognitive or behavioral impairment [3], and around 5 to 15% have cognitive and behavioral changes that fulfill the diagnostic criteria for frontotemporal dementia (FTD) [4–6].

Cognitive impairment may influence patient survival [7, 8], increase caregiver burden [8, 9], and affect decision-making processes during treatment [10]. Hence, it is of great importance to accurately identify the cognitive status of ALS patients. At present, this process largely relies on comprehensive neuropsychological assessment [11]. In ALS patients, studies have detected deficits in various cognitive domains including executive function, social cognition, language, and working memory [11]. It is time-consuming to assess all the domains comprehensively via non-ALS-specific neuropsychological batteries [12]. Moreover, many screening tools are inappropriate for patients with severe physical deficits in speech, writing, and drawing [12, 13]. Therefore, the Edinburgh cognitive and behavioral screen (ECAS), a rapid neuropsychological screening tool, was specifically developed to identify cognitive and behavioral changes of ALS patients [13]. Remarkably, ECAS has been widely implemented in both clinical and research settings with different language versions and has proven to be an efficient and reliable screening tool for ALS patients [12, 14–18].

Previous studies have investigated the changes in peripheral blood lymphocyte subsets in ALS patients [19–25]. Studies showed significantly higher [19] or similar [20] level of T lymphocyte, similar level of B lymphocyte [19, 20], and significant increase [19, 21, 22] or no change [20] in the number of NK cell in ALS patients compared to healthy controls. While most studies found increased percentage of CD4+ T lymphocyte in ALS patients [19, 20, 23], several studies found decrease [24] or no change [21] in CD4+ T lymphocyte. Level of CD8+ T lymphocyte was found to be increased [19, 22], reduced [20, 25], or unchanged in ALS patients [21, 23]. Although these studies did not provide conclusive results, they strongly supported changes in the peripheral immunity in ALS patients.

Importantly, changed peripheral immunity was found to be related to cognitive impairment of patients with various neurodegenerative diseases, such as Alzheimer’s disease (AD) [26, 27] and Parkinson’s disease (PD) [28, 29]. Nevertheless, there has been no study exploring the relationship between the peripheral blood lymphocyte subsets and the cognitive status in ALS patients.

In this study, we evaluated the peripheral blood lymphocyte subsets in ALS patients with and without cognitive impairment, and analyzed the predictive value of lymphocyte subsets for cognitive impairment in ALS patients.

## Methods

### Participants

From April 2018 to October 2020, patients who visited the Department of Neurology, Tongji Hospital, Wuhan and who were diagnosed with possible, probable, or definite ALS according to the revised El Escorial criteria [30] were included in our study. Exclusion criteria included a history of autoimmune diseases or hematological disorders, concurrent infectious diseases, use of immunosuppressive agents, anti-inflammatory medications or corticosteroids that might affect the function of the immune system, and other neurological diseases such as cerebrovascular diseases, epilepsy, traumatic brain injury, and dementia. Additionally, none of the ALS patients enrolled since the start of the pandemic had a history of COVID-19. Our study was approved by the Ethics Committee of Tongji Hospital (TJ-IRB20201219), and all participants signed written informed consent before the enrollment.

### Demographic and clinical data acquisition

Demographic data including gender, age at testing, age at onset, disease duration and education time were collected during the patient visit. The cognitive function of patients was evaluated with the Chinese version of ECAS [12], using a cut-off ECAS total score of 81.92 out of 136 calculated as the mean value subtracted by two standard deviations obtained by the healthy Chinese population, where several subdomains additionally underwent Chinese language specific modifications [12]. ALS patients were consequently divided into the group of patients with cognitive impairment (ALS-ci) and the group of patients without cognitive impairment (ALS-nci). Behavioral impairment in ALS patients was defined as at least one behavioral abnormality determined by the behavioral part of ECAS. The severity of physical disability was evaluated by the ALS Functional Rating Scale-Revised (ALSFRS-R) [31].

### Flow cytometry

Venous blood sampling was performed between 5:00 a.m. and 7:00 a.m. during the hospital stay for each patient. Peripheral blood lymphocyte subsets including peripheral total T lymphocyte, CD4+ T and CD8+ T lymphocyte, B lymphocyte, and NK cell were quantified by flow cytometry (flow cytometer: BD FACSCantoTM II; antibodies: BD Multitest™ 6-color TBNK) based on their forward scattering characteristics and lateral scattering characteristics after immunostaining. Results of each lymphocyte subset were expressed as absolute number (/μL) as well as percentage value divided by total lymphocyte (%). For T lymphocyte, CD4+ /CD8+ ratio was also calculated.

### Statistical analysis

Shapiro-Wilk test was used to determine the distribution of continuous data. To compare the parameters between the two groups, independent t-test was used for normally distributed continuous data that were expressed as mean ± standard deviation and Mann-Whitney U test was used for non-normally distributed continuous data that were expressed as median [interquartile range]. Chi-square test was used for categorical data that were reported as frequencies.

Demographic, clinical and lymphocyte parameters were examined using univariate regression analysis for the prediction of cognitive impairment in ALS patients. Significant parameters were further included as independent variables in a multivariate logistic analysis with cognitive impairment as a dependent variable. Independent continuous variables that were not normally distributed were transformed into normalized data when possible. Predictive power of these parameters was assessed via the area under the receiver operating characteristic (ROC) curve value with sensitivity and specificity values calculated based on the maximal Youden Index. Pearson correlation or Spearman correlation analysis was performed between each significant independent variable from the multivariate regression analysis and the ECAS total score in case of normal or non-normal data distribution. All statistical analyses were performed using the SPSS Software (version 25.0) with the significant threshold set as *p* < 0.05.

## Results

### Demographic and clinical characteristics

Eighty-one ALS patients were enrolled in this study (age 54.9 ± 11.2 years; 48 males). Demographic and clinical characteristics of the participants were summarized in Table 1. Of all ALS patients, mean age at onset was 54.2 ± 11.1 years, median disease duration was 12 months (ranging from 2 to 64 months), and median ALSFRS-R score was 42 (ranging from 15 to 48). 64 (79.0%) patients had limb onset, 15 (18.5%) patients had bulbar onset, and 2 (2.5%) patients had mixed onset (Table 1).

**Table 1:** Demographic and clinical characteristics of ALS patients

	ALL (81 patients)	ALS-nci (49 patients)	ALS-ci (32 patients)	*P* value
Male	48 (59.3%)	30 (61.2%)	18 (56.3%)	0.656
Education time (years)	9 [9–12]	12 [9–12]	7.5 [6–9]	**< 0.001**
Age at onset (years)	54.3 ± 11.1	51.0 ± 10.9	59.3 ± 9.6	**< 0.001**
Age at testing (years)	54.9 ± 11.2	51.6 ± 10.9	60.1 ± 9.6	**< 0.001**
Disease duration (months)	12 [7–18]	11 [6–16]	14 [9–21]	**0.025**
BMI (kg/m^2^)	22.10 ± 2.94	22.36 ± 3.22	21.70 ± 2.44	0.368
Site of onset				0.430
Bulbar	15	7	8	
limb	64	41	23	
mixed	2	1	1	
ALSFRS-R score	42 [36.5–45]	42 [36–46]	39 [37–43.5]	0.309

*Abbreviations*: *ALS-nci* ALS without cognitive impairment, *ALS-ci* ALS with cognitive impairment, *BMI* Body mass index

32 (39.5%) ALS patients had cognitive impairment (ALS-ci) and 49 (60.5%) ALS patients had no cognitive impairment (ALS-nci). Two patients in the ALS-ci group and three patients in the ALS-nci group did not have the behavioral part of ECAS since they were neither accompanied by caregivers during hospitalization nor had they close contacts during post-hospitalization call. 3 (10%) patients had disinhibition, 6 (20.0%) patients had apathy, 5 (16.7%) patients had loss of sympathy, 1 (3.3%) patient had perseveration, and 2 (6.7%) patients had changes in eating behaviour in the ALS-ci group, while 1 (2.2%) patient had disinhibition, 4 (8.7%) patients had apathy, 4 (8.7%) patients had loss of sympathy, 2 (4.3%) patients had perseveration, and 2 (4.3%) patients had changes in eating behaviour in the ALS-nci group. Behavioral abnormalities did not differ between the two groups (Supplemental Table 1).

Patients from the ALS-ci group had shorter education time (9 years vs. 12 years, *P* < 0.001), older age at testing (60.1 years vs. 51.6 years, *P* < 0.001), as well as at symptom onset (59.3 years vs. 51.0 years, *P* < 0.001), and longer disease duration (14 months vs. 11 months, *P* = 0. 0.025) than those in the ALS-nci group. Gender ratio, BMI values, site of onset, and ALSFRS-R scores did not differ significantly between the two groups (Table 1).

### Changes in the peripheral blood lymphocyte subsets

Compared to the ALS-nci group, patients in the ALS-ci group had significantly lower numbers of total T lymphocyte (996.50/μL vs 1247.00/μL, *P* = 0.005), CD4+ T lymphocyte (652.47/μL vs. 767.63/μL, *P* = 0.019), CD8+ T lymphocyte (262.00/μL vs. 360.00/μL, *P* = 0.019), and B lymphocyte (158.50/μL vs. 219.00/μL, *P* = 0.035). Number of NK cell, percentages of total T lymphocyte, CD4+ T lymphocyte, CD8+ T lymphocyte, B lymphocyte, and NK cell, and CD4+/CD8+ ratio were similar between the two groups (*P* > 0.05) (Table 2).

**Table 2:** Comparison of peripheral blood lymphocyte subsets between the ALS-ci group and the ALS-nci group

Lymphocyte subsets	ALS-nci (49 patients)	ALS-ci (32 patients)	*P* value
Total T (#)	1247.00 [962.50–1375.00]	996.50 [785.50–1149.50]	**0.005**
CD4+ T (#)	767.63 ± 224.23	652.47 ± 190.75	**0.019**
CD8+ T (#)	360.00 [277.00–466.00]	262.00 [210.00–432.50]	**0.019**
B (#)	219.00 [144.00–297.50]	158.50 [136.25–213.00]	**0.035**
NK (#)	179.00 [132.00–284.50]	194.00 [115.50–376.75]	0.896
Total T (%)	73.36 [68.06–76.55]	68.97 [63.80–78.74]	0.284
CD4+ T (%)	45.75 ± 7.94	44.79 ± 8.06	0.598
CD8+ T (%)	22.75 ± 6.24	21.03 ± 7.92	0.309
B (%)	13.60 ± 4.76	12.89 ± 4.97	0.519
NK (%)	11.51 [9.32–18.54]	13.96 [8.91–20.74]	0.356
CD4+/CD8+ ratio	2.01 [1.59–2.73]	2.15 [1.69–3.47]	0.484

Significant *P* values were shown in bold

*Abbreviations*: *ALS-nci* ALS without cognitive impairment, *ALS-ci* ALS with cognitive impairment. (#) = cell count of each lymphocyte subset; (%) = lymphocyte subset count/ total lymphocyte count

### The predictive model of cognitive impairment in ALS patients

**Fig. 1:**
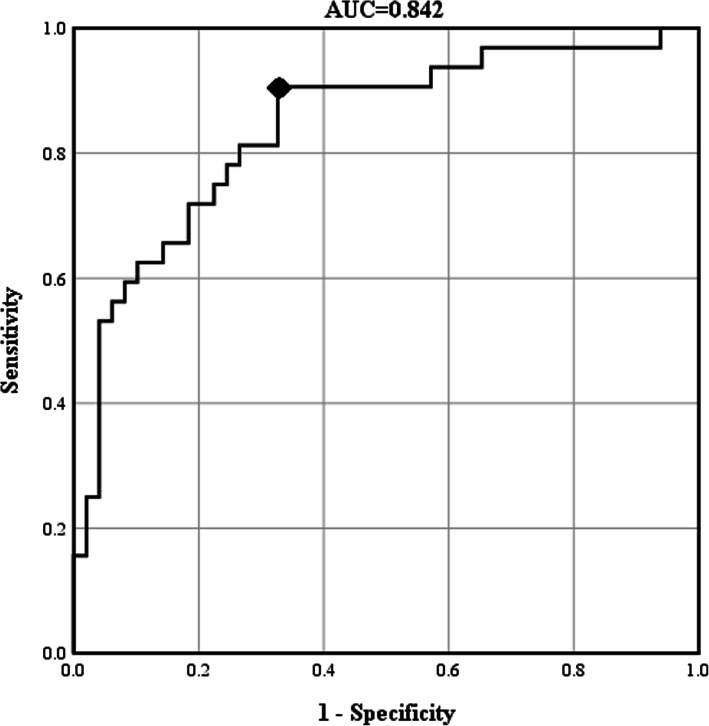
ROC curve for cognitive impairment in ALS patients. Legend: The area under the curve was 0.842 (95% CI 0.775–0.933, *P* < 0.001), with a sensitivity of 90.6% and a specificity of 67.3%.

Univariate logistic regression showed that older age at onset (OR 1.030, 95% CI 1.085–1.143, *P* = 0.002) or at testing (OR 1.090, 95% CI 1.034–1.149, *P* = 0.001), education time less than 9 years (OR 5.318, 95% CI 1.859–15.215, *P* = 0.002), longer disease duration (OR 1.769, 95% CI 1.072–2.919, *P* = 0.026), and higher levels of total T lymphocyte (OR 0.530, 95% CI 0.318–0.882, *P* = 0.015), CD4+ T lymphocyte (OR 0.997, 95% CI 0.995–0.999, *P* = 0.024), and CD8+ T lymphocyte (OR 0.582, 95% CI 0.355–0.955, *P* = 0.032) were significant predictors of cognitive impairment of ALS patients (Tables 3 and 4). Only older age at testing (OR 1.107, 95% CI 1.041–1.177, *P* = 0.001), education time less than 9 years (OR 6.995, 95% CI 2.068–23.663, *P* = 0.002), and lower level of CD4+ T lymphocyte (OR 0.997, 95% CI 0.995–0.999, *p* = 0.049) remained significant in multivariate logistic regression analysis (Table 5). The model combining older age at testing, lower education level, and lower number of CD4+ T lymphocyte predicted a higher risk of cognitive impairment of ALS patients, with an area under the ROC curve value of 0.842 (95% CI 0.775–0.933, *P* < 0.001), a sensitivity of 90.6%, and a specificity of 67.3% (Fig. 1).

**Table 3:** Univariate logistic regression by including demographic and clinical parameters

	OR	95% CI	*P*
Gender			0.656
Male	1		
Female	1.228	(0.497–3.034)	
Education time			**0.002**
>9 years	1		
≤9 years	5.318	(1.859–15.215)	
Age at onset	1.030	(1.085–1.143)	**0.002**
Age at testing	1.090	(1.034–1.149)	**0.001**
Disease duration^a^	1.769	(1.072–2.919)	**0.026**
BMI	0.925	(0.781–1.095)	0.363
Site of onset			0.449
Bulbar	1		
Limb	0.491	(0.158–1.528)	
Mixed	0.875	(0.046–16.744)	
ALSFRS-R score^a^	0.783	(0.490–1.249)	0.304

Significant P values were shown in bold

*Abbreviations*: *BMI* Body mass index

^a^These parameters were transformed into normally distributed data for logistic regression analysis

**Table 4:** Univariate logistic regression by including lymphocyte parameters

Lymphocyte subsets	OR	95% CI	P
Total T (#) ^a^	0.530	(0.318–0.882)	**0.015**
CD4+ T (#)	0.997	(0.995–0.999)	**0.024**
CD8+ T (#) ^a^	0.582	(0.355–0.955)	**0.032**
B (#) ^a^	0.660	(0.409–1.065)	0.089
NK (#) ^a^	0.934	(0.593–1.472)	0.768
Total T (%) ^a^	0.778	(0.490–1.237)	0.289
CD4+ T (%)	0.985	(0.931–1.042)	0.593
CD8+ T (%)	0.966	(0.905–1.032)	0.305
B (%)	1.969	(0.882–1.065)	0.514
NK (%) ^a^	1.275	(0.803–2.024)	0.304
CD4+ /CD8+ ratio ^a^	1.199	(0.758–1.897)	0.438

(#) = cell count of each lymphocyte subset; (%) = lymphocyte subset count/ total lymphocyte count

Significant *P* values were shown in bold

^a^These parameters were transformed into normally distributed data for logistic regression analysis

**Table 5:** Multivariate logistic regression analysis for the prediction of cognitive impairment in ALS patients

Variables	OR	95% CI	P value
Age at testing	1.107	(1.041–1.177)	**0.001**
Education time			
>9 years	1		
≤9 years	6.995	(2.068–23.663)	**0.002**
CD4+ T (#)	0.997	(0.995–0.999)	**0.049**

CD4+ T (#): cell count of CD4+ T lymphocyte

Significant *P* values were shown in bold

### Correlation analysis between demographic, clinical, and lymphocyte parameters and the ECAS total score

Significant predictors of cognitive impairment in the multivariate regression analysis were further examined by correlation analysis to explore their relationship with the ECAS total score. There was a negative correlation between age at testing and the ECAS total score (*r* = − 0.397, *P* < 0.001) and a positive correlation between education time and the ECAS total score (*r* = 0.691, *P* < 0.001), while no significant correlation between the number of CD4+ T lymphocyte and the ECAS total score was found (*r* = 0.180, *P* = 0.107).

## Discussion

Our study showed that ALS patients with cognitive impairment (ALS-ci group) displayed a different peripheral immune profile compared to those without (ALS-nci group). The numbers of total T lymphocyte, CD4+ T lymphocyte, CD8+ T lymphocyte, and B lymphocyte were all decreased in the ALS-ci group. Although the number of CD4+ T lymphocyte was not correlated with the ECAS total score, it was a significant predictor of cognitive impairment in ALS patients along with older age at testing and lower education level. To the best of our knowledge, this is the first study investigating the relationship between peripheral blood lymphocyte subsets and cognitive status in ALS patients.

In our patients, we found significant reduction of total T lymphocyte, CD4+ T and CD8+ T lymphocyte, and total B lymphocyte in the ALS patients with cognitive impairment compared to those without. Additionally, lowered number of CD4+ T lymphocyte seemed to be an independent risk factor of cognitive impairment in ALS patients. Alterations of the peripheral immune system have been reported to be related to cognitive impairment in other neurodegenerative disease [26–29]. Reduction of B and T lymphocytes was detected in patients with dementia of different patterns including AD, vascular dementia, and FTD [26]. One study found that the number of CD4+ T lymphocyte was positively associated with MMSE scores in AD patients [27]. In PD patients with cognitive impairment, Hu et al. detected significant lower numbers of CD4+, CD8+, and total T lymphocyte, and decreased CD4+/CD8+ T ratio [28]. Magistrelli et al. found that PD patients with cognitive impairment had increased activated regulatory T-lymphocyte (Treg) and Th1 lymphocytes [29]. The imbalance between CD4+ T lymphocyte subpopulations may lead to a proinflammatory state with an overproduction of proinflammatory cytokines that could impair the blood-brain barrier, reach the central nervous system (CNS), and further aggravate neurodegeneration [32]. Thus, it is possible that a fine-tuned balance of immune cells plays a major role in both the intactness of blood-brain barrier [33] as well as the maintenance of cross-talk between immune cells, glia and neurons [34], and abnormal peripheral immunity might be related to pathophysiological processes of cognitive impairment in different neurodegenerative diseases.

Recent basic studies might provide some clues on the periphery immune abnormality in ALS patients with cognitive impairment. Animal studies indicated that adaptive immunity affects cognitive performance, and T lymphocytes are the major immune players in this process [35–37]. Early work demonstrated that mice with severe combined immune deficient (SCID, deficient in both T cell and B cell responses) performed poorly in spatial learning and memory tasks compared with wild type (WT) mice [35, 36], while cognitive deficit can be reversed by reconstituting the T lymphocyte compartment in SCID mice [35]. Additionally, depletion of adaptive immunity also led to impaired learning behavior in WT mice, while cognitive dysfunction following immunity ablation could be restored by passive transfer of autologous T lymphocytes in WT mice [35]. Interestingly, learning behavior was not impaired in mice that were specifically depleted of B lymphocytes [37]. While the blood-brain barrier efficiently precludes lymphocyte entry into the brain parenchyma, a large number of T lymphocytes reside in the meninges and are considered to influence the brain function [38]. Mice exhibit learning deficits when T cell migration was blocked from the peripheral blood to the meninges [38]. These studies suggested that the deficit of peripheral T lymphocytes could lead to cognitive impairment. In comparison, disruption of the blood-brain barrier that could permit the entry of proinflammatory cells to the CNS were identified both in ALS animal models [39–41] and ALS patients [39, 42–44]. In particular, T lymphocytes were found to contribute to the neuro-inflammatory processes and infiltrate the CNS during disease progression of ALS [45]. However, the role of T lymphocytes in cognitive impairment might be different from its role in the neuro-degenerating processes in ALS patients.

Our study had several limitations. The study had a cross-sectional design and a small sample size. Thus, caution should be raised when interpreting the “predictors” of cognitive impairment identified by the logistic regression analysis performed in our study. In addition, detailed subsets of peripheral blood lymphocytes, including naïve T, memory T, and Treg lymphocytes were not evaluated in our study. While the revised Strong criteria [6] helps classify ALS patients with cognitive or/and behavioral impairment, our methodology only compared the percentage of patients with behavioral impairment in the ALS-ci group and the ALS-nci group based on the behavioral part of ECAS and was insufficient to categorize ALS patients set by the revised Strong criteria [6]. Additionally, cut-off scores for behavioral abnormalities defined in ECAS might need further exploration and more studies that fulfill the methodology requirements set by the revised Strong criteria are needed to determine whether changed immunity is related to behavioral abnormalities in ALS patients. Importantly, the C9orf72 gene mutation, which is strongly related to ALS/FTD and could impact the cognitive status [46], was not examined in our study, although it had been shown that frequency of C9orf72 mutation in the Chinese population is rather low [47]. Moreover, since all enrolled participants are Chinese and the genetic contribution to innate immunity is not clearly known, caution is warranted when findings are extrapolated to the general ALS population. Furthermore, emotional [48], respiratory [49], metabolic factors [50–52] were not evaluated in our study.

## Conclusion

A relationship between the peripheral immune changes and the occurrence of cognitive decline in ALS patients was identified. In particular. ALS patients with a reduced peripheral CD4+ T lymphocyte level may be more vulnerable to cognitive impairment apart from aging processes and lower educational level. Further studies are required to investigate the pathophysiological mechanisms underlying this observation.

## Supplementary Information

**Additional file 1.**

## Data Availability

Data are preserved locally and are available from the corresponding author on reasonable request.

## Abbreviations

AD: Alzheimer’s diseas
ALS: Amyotrophic lateral sclerosis
ALSFRS-R: The ALS Functional Rating Scale-Revised
CNS: Central nervous system
ECAS: Edinburgh cognitive and behavioral screen
FTD: Frontotemporal dementia
PD: Parkinson’s disease
ROC: Receiver operating characteristic curve
SCID: Severe combined immune deficient
Treg: Regulatory T lymphocyte
WT: Wild type
