# Supplementary material for: Decreased blood CD4+ T lymphocyte helps predict cognitive impairment in patients with amyotrophic lateral sclerosis
Source: BMC Neurol. 2021 Apr 12;21:157. doi: 10.1186/s12883-021-02185-w (PMC8039093; doi:10.1186/s12883-021-02185-w)
Supplement: Supplementary file 1 — Additional file 1. [file 12883_2021_2185_MOESM1_ESM.docx]

| **Supplemental Table 1. Numbers of ALS patients with behavioral subdomain abnormalities** | | | |
| --- | --- | --- | --- |
| Behavioral subdomains | ALS-nci  (46 patients) | ALS-ci  (30 patients) | P value |
| Disinhibition | 1 | 3 | 0.850 |
| Apathy | 4 | 6 |  |
| Loss of sympathy | 4 | 5 |  |
| Perseveration | 2 | 1 |  |
| Changes in eating behavior | 2 | 2 |  |
| Abbreviations: ALS-nci: ALS without cognitive impairment; ALS-ci: ALS with cognitive impairment. | | | |
